# Experimental validation of a new biphasic model of the contact mechanics of the porcine hip

**DOI:** 10.1177/0954411914537618

**Published:** 2014-06

**Authors:** Junyan Li, Qianqian Wang, Zhongmin Jin, Sophie Williams, John Fisher, Ruth K Wilcox

**Affiliations:** 1 Institute of Medical and Biological Engineering, School of Mechanical Engineering, University of Leeds, Leeds, UK; 2 School of Mechanical Engineering, Xi’an Jiaotong University, Xi’an, China

**Keywords:** Cartilage, biphasic, hip, contact mechanics, hemiarthroplasty, finite element

## Abstract

Hip models that incorporate the biphasic behaviour of articular cartilage can improve understanding of the joint function, pathology of joint degeneration and effect of potential interventions. The aim of this study was to develop a specimen-specific biphasic finite element model of a porcine acetabulum incorporating a biphasic representation of the articular cartilage and to validate the model predictions against direct experimental measurements of the contact area in the same specimen. Additionally, the effect of using a different tension–compression behaviour for the solid phase of the articular cartilage was investigated. The model represented different radial clearances and load magnitudes. The comparison of the finite element predictions and the experimental measurement showed good agreement in the location, size and shape of the contact area, and a similar trend in the relationship between contact area and load was observed. There was, however, a deviation of over 30% in the magnitude of the contact area, which might be due to experimental limitations or to simplifications in the material constitutive relationships used. In comparison with the isotropic solid phase model, the tension–compression solid phase model had better agreement with the experimental observations. The findings provide some confidence that the new biphasic methodology for modelling the cartilage is able to predict the contact mechanics of the hip joint. The validation provides a foundation for future subject-specific studies of the human hip using a biphasic cartilage model.

## Introduction

The hip joint is one of the most heavily loaded joints in the human body. The unique biphasic properties of the articular cartilage on the bearing surfaces of the joint are critical to its longevity because the solid matrix in the cartilage can be protected from a high proportion of external loads through interstitial fluid pressurisation.^1–5^ It is necessary, therefore, to consider the biphasic properties of the cartilage within the joint to better understand the joint performance and how changes in these properties may lead to joint degeneration and the need for potential intervention.

In an experimental setting, the measurements that can be taken to characterise the contact behaviour of the natural hip joint are limited.^6–8^ For this reason, many investigators have adopted a computational approach, often using ABAQUS (Dassault Systèmes, Suresnes Cedex, France) to simulate the biphasic behaviour of the cartilage in a finite element (FE) model.^9–13^ However, this method is not generally suitable for dealing with biphasic cartilage-on-cartilage contact under high physiological loads or over prolonged loading periods because there are difficulties in obtaining convergence. Our recent research has overcome these issues by employing an open-source solver, FEBio (mrl.sci.utah.edu/software/febio), which substantially improves convergence for biphasic models of whole joints.^14^ Prior to applying this method to clinical studies, it is first important to evaluate the accuracy of the technique and validate the model predictions.

The cartilage layer is an inhomogeneous fibre-reinforced structure.^4,15,16^ Previous studies using models of cylindrical cartilage sections have demonstrated that the constitutive relationship proposed by Ateshian’s group, in which the tensile and compressive behaviour are different, captures the mechanical performance of the tissue more realistically than an isotropic relationship.^17–19^ Limited applications to the knee joint model have been made,^11–13^ but the influence of this more sophisticated material model on the contact mechanics of the whole hip joint has yet to be evaluated.

The aim of this study was therefore to develop a specimen-specific biphasic FE model of a porcine hip following hemiarthroplasty and validate the predictions of the recently published biphasic model^14^ against direct experimental measurements of the contact area. Additionally, the contribution of a different tension–compression (T-C) modulus for the solid phase of the cartilage in the hip was evaluated.

## Methods

### Experimental measurement of contact area

An acetabulum was dissected carefully from a 2.4-year-old pig and all soft tissues except for the cartilage were removed to facilitate the geometry reconstruction in the FE model. The acetabulum was kept hydrated by phosphate-buffered saline constantly during the tissue preparation. It was first imaged using a micro computed tomography (µCT) scanner (µCT 80; Scanco Medical AG, Brüttisellen, Switzerland) at a cubic voxel size of 73.6 µm and energy of 70 kVp, 114 µA.

**Figure 1.**
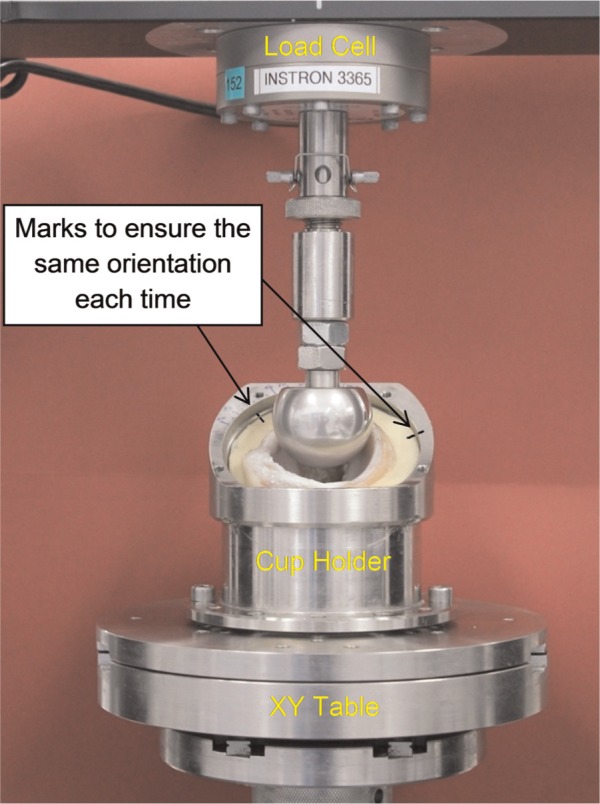
Experimental set-ups of hemiarthroplasty hip joint in the material testing machine Instron model 3365. The X–Y table allowed the acetabulum to translate in the horizontal plane under compressive loading. Femoral metal head moved along the vertical direction to produce the demand load.

The acetabulum was then loaded against a prosthetic femoral head (i.e. a hemiarthroplasty) under a number of static single-axis loading conditions and the contact area was determined. Other parameters, such as the contact pressure, were not measured experimentally because of the highly conforming nature of the joint and the potential to introduce substantial measurement artefacts if flat transducer films were introduced into the hemispherical joint space. The tests were undertaken using a materials testing machine (Model 3365; Instron Ltd, High Wycombe, UK). In the loading frame, the acetabulum was inverted and fixed in a cup holder with polymethyl methacrylate cement (W.H.W Plastics, North Humberside, UK). The cup holder provided an inclination angle of 35° (equivalent to 45° in vivo) and was fitted on an X–Y table, which allowed self-alignment by enabling free translation in the horizontal plane (Figure 1). Two sizes of spherical cobalt chrome heads with diameters of 37 and 40 mm were used to produce contact conditions at different radial clearances. The joint was loaded under compression by applying loads of 10, 50, 100, 200 and 400 N to the metal head. The loads were ramped up over 10 s by controlling the loading speed. The maximum loading was estimated from other quadrupeds,^20,21^ and while this and the loading rate may not replicate the in vivo situation, they were considered appropriate here since the primary purpose was validation of the FE model. Initially, the head surface was coated by a thin layer of fluid polymer (101RF; Microset Products Ltd, Leicestershire, UK) and the acetabular cartilage was kept clean. After each loading case, the specimen was unloaded and photographs of the head and acetabulum were taken using a high-definition digital camera (EOS 550D; Canon Inc., Tokyo, Japan). The area in contact was identified by both the stained surface of the cartilage and the pattern change on the surface of head. The two-dimensional contact area displayed in the photographs of the metal head was analysed in a professional imaging package (Image-Pro version 6.3; Media Cybernetics, Rockville, MD, USA) and was projected onto a three-dimensional spherical surface in SolidWorks (version 19.4; Dassault Systèmes SolidWorks Corpora-tion, Massachusetts, USA) to give the magnitude of the contact area. The measurement was repeated three times for each load condition and the mean value taken. For each measurement, fresh polymer was applied to the metal head. Lines were marked on the cup holder and cement block to ensure that the orientation of acetabulum was the same for each measurement and during photographing. The measurement accuracy was assessed using a total hip replacement (ceramic head against ultra-high-molecular-weight polyethylene cup) and comparing the measured contact area to the analytically calculated area. The average difference between the experimentally measured contact radius and the analytical solution was 12% across the loading cases studied. The experimental measurement underestimated the analytical solution at higher loads. In terms of contact area, this equated to the experimentally measured area being 22%−35% lower than the analytical value when the contact areas were similar to the largest area found in this study (i.e. 400 N on 40 mm head).^22^ The acetabular tissue was allowed to recover for about 10 min between loading conditions. This recovery period was deemed adequate because the loading period was too short to cause evident cartilage consolidation, as found in a previous study.^14^

### FE modelling

**Figure 2.**
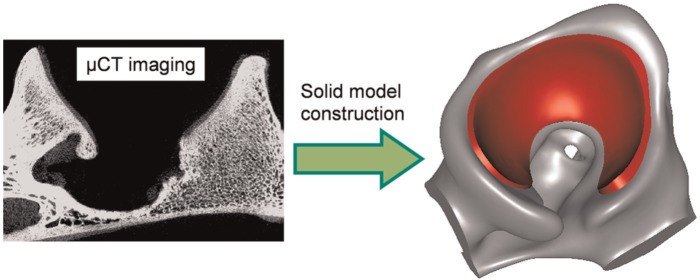
Three-dimensional solid model constructed in Geomagic from µCT imaging, illustrating the bone (grey) and the cartilage (red). Both the bone and the cartilage were displayed very vividly in the µCT images, and highly accurate model geometry was achieved. µCT: micro computed tomography.

The volumetric micro CT data in DICOM format were imported into an image processing and meshing software package (ScanIP version 5.1; Simpleware Ltd, Exeter, UK) for segmentation and smoothing. A typical image slice is shown in Figure 2. The bone and the whole acetabulum including both the subchondral bone and the cartilage were identified sequentially by greyscale thresholding. The surface of the bone model and the whole acetabular model were meshed with three-noded triangular elements and exported in STL format into another surface-generation software package (Geomagic Studio 11, Geomagic Inc., Research Triangle Park, NC, USA). Boolean algorithms were performed to exclude the bone model from the whole acetabular model that included both the subchondral bone and the cartilage in order to obtain a model representing just the cartilage (Figure 2).

**Figure 3.**
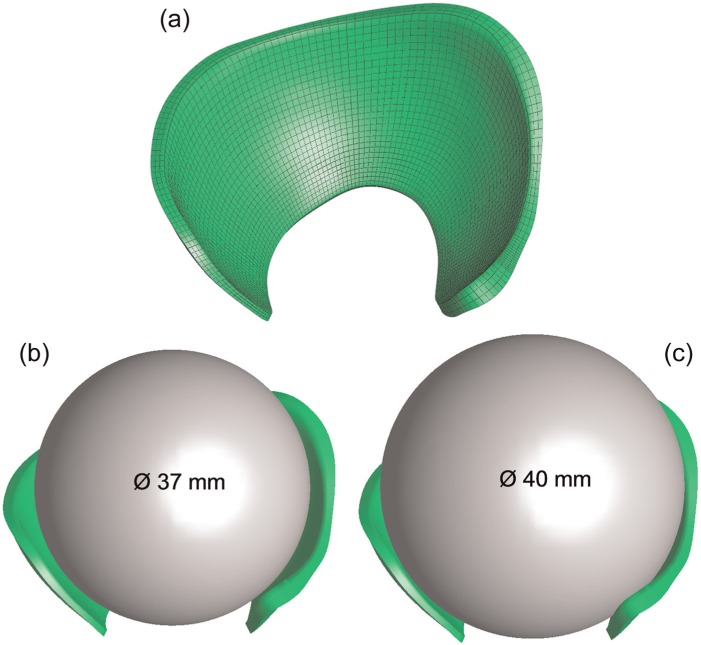
FE model creation: (a) the cartilage represented by hexahedral elements. (b, c) FE models of hemiarthroplasty with heads of two different dimensions.

The general approach to modelling the natural hip using FEBio (version 1.5.0; Musculoskeletal Research Laboratories, Salt Lake City, UT, USA; mrl.sci.utah.edu/software/febio) was described previously.^14^ For this specimen-specific model, the cartilage surfaces were reconstructed from tens of thousands of triangles into several patches to form a solid model which was then imported into ABAQUS (version 6.11-1; Dassault Systèmes) for meshing. The FE model of the cartilage was composed of 9906 eight-noded hexahedral elements. The prosthesis heads used in the experiment were represented by two spheres with diameters of 37 and 40 mm (Figure 3). The spherical head was meshed with 7800 eight-noded hexahedral elements that were rigidly constrained to a reference point. The bone was assumed to be rigid and therefore was not included in the FE model. Mesh sensitivity studies were performed to ensure that a doubling of the element number changed the outputs of interest by less than 5%.

The meshed model was then imported into PreView (version 1.7; mrl.sci.utah.edu/software.php) for pre-processing. The acetabular cartilage was oriented and positioned according to the experimental set-up. The surface of the cartilage that was connected to the subchondral bone was rigidly constrained to a reference point, which was fixed in all the degrees of freedom in order to represent the rigid bone. The spherical head was assumed to be impermeable. The contact was assumed to be frictionless, and the fluid flow on the articulating surface of the cartilage was considered as contact dependent so that fluid could only flow out from the area of the articulating surface that was not in contact with the impermeable head. To replicate the constraints of the head relative to the acetabular cartilage, the five loads were applied over a 3-s period to the rigid head, which was fixed along rotational degrees of freedom. The shorter loading period of the FE models than the experiment was to enhance the computational efficiency but did not affect the model prediction, because there was almost no time-dependent response of the hip for a period of less than 10 s.^14^

The cartilage was treated as a biphasic solid. Both an isotropic solid phase and a T-C solid phase were studied to evaluate the effect of different constitutive relationships on the model predictions of contact area. The solid phase of the isotropic models was represented by the neo-Hookean constitutive relationship with the properties adopted from a previous study (Table 1).^10^ Additionally, the isotropic model was run with twice the previously defined aggregate modulus^10^ to investigate the sensitivity to this value. For the T-C model, the compressive aggregate modulus was the same as the isotropic model and the tensile modulus was set to 10 times higher.^17,19^ The higher tensile modulus was implemented by incorporating a fibre material into the isotropic material. The fibre material had three orthogonal fibre directions with a linear constitutive relationship and continuous spatial distribution. The fibres only sustain tension, so that the material as a whole is isotropically and homogeneously fibre-reinforced.

**Table 1.** Material properties for the models with different solid phase properties.

	Isotropic	2E	T-C
Aggregate modulus (MPa)	0.562	1.124	0.562
Tensile modulus (MPa)	N/A	N/A	5.62
Poisson’s ratio	0	0	0
Permeability (mm^4^/N s)	0.00157	0.00157	0.00157

Isotropic: the model with isotropic solid phase; 2E: the isotropic model with doubled aggregate stiffness; T-C: the model with T-C solid phase.

The FE simulations were conducted using FEBio (version 1.6.0) on a Linux server with 8 GB of RAM and 8 Intel X5560 cores at 2.8 GHz. The contact stress was recorded and the contact area was calculated by summing the area of the articulating surface elements in which the contact stress was non-zero. The magnitude, location and shape of the contact area were compared between the experimental measurements and the model predictions. The location and shape were compared by projecting the outline of the experimentally measured contact region onto the model output.

## Results

**Figure 4.**
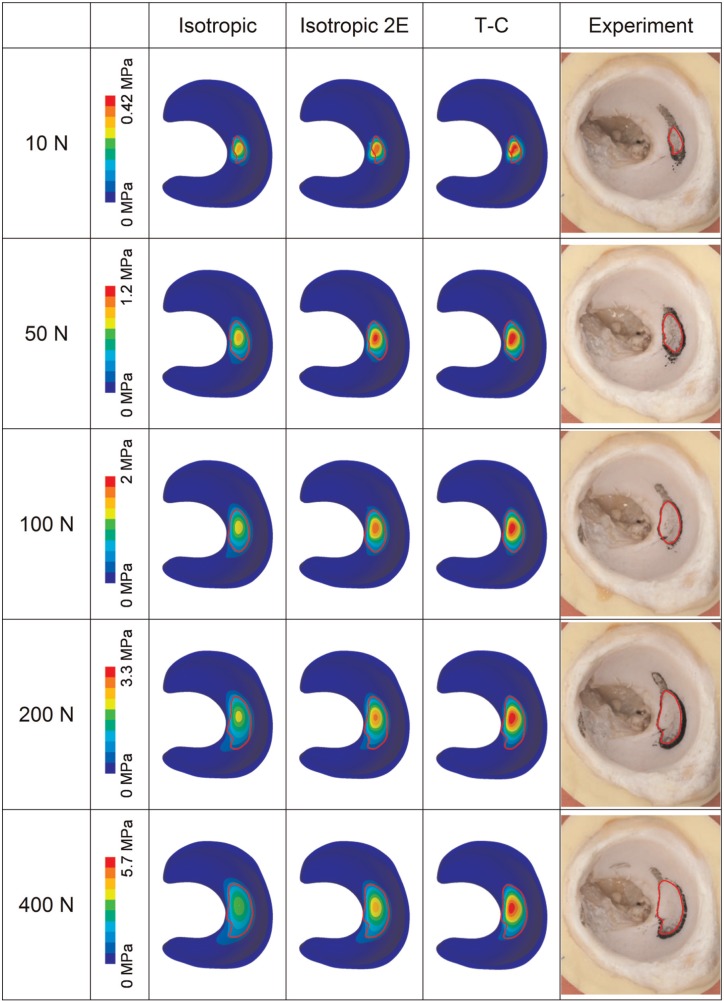
Contours of contact stress of the FE models (head diameter = 37 mm) with different solid phase properties in comparison to the experimentally measured contact area (polymer mark in black). The edge of the experimentally measured contact area was outlined in red and projected to the models for comparison. The contact stress contour instead of the pure contact area of the models is presented because it not only exhibits the area in contact but also facilitates the comparison in contact stress for the models with different material properties. T-C: tension–compression.

**Figure 5.**
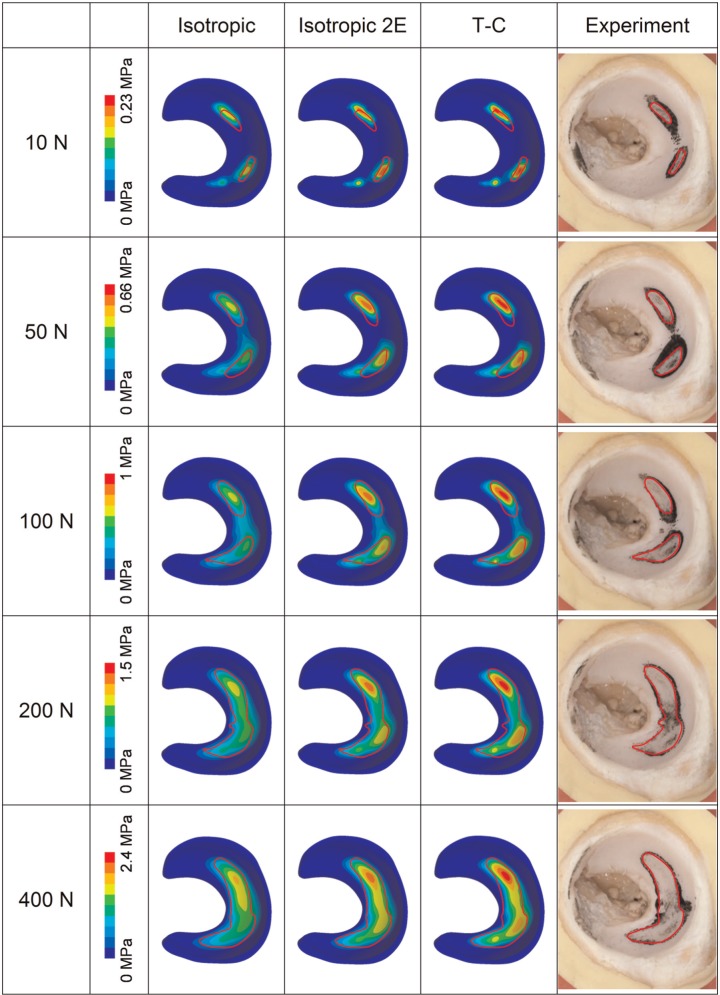
Contours of contact stress of the FE models (head diameter = 40 mm) with different solid phase properties in comparison to the experimentally measured contact area (polymer mark in black). T-C: tension–compression.

The contours of contact stress of the FE models and the experimentally measured contact area for the 37- and 40-mm femoral head diameter cases are shown in Figures 4 and 5, respectively. Generally, the location and shape of the contact area for the models with different solid phase properties were similar. The model with the isotropic solid phase had larger contact area and 30%−40% lower peak contact stress (under the load of 400 N), as compared to the model with the T-C solid phase. However, comparable magnitudes of contact stress and contact area were found between the T-C model and the isotropic model with doubled stiffness. For both femoral head dimensions, good agreement in the location, shape and area of the contact was found between the FE models and the experimental measurement over the range of loads investigated. In terms of the shape and area of the contact, the T-C model was more comparable to the experimental measurement than the isotropic model. Particularly, in the case of the 40-mm head diameter (Figure 5), the contact, as measured experimentally, occurred in two separate locations under loads from 10 to 100 N and merged into one region for the loads of 200 and 400 N. This pattern was observed for the FE model with T-C solid phase and the isotropic model with doubled aggregate stiffness. However, in the isotropic model, the two separate contact locations joined together at loads of 100 N or greater.

**Figure 6.**
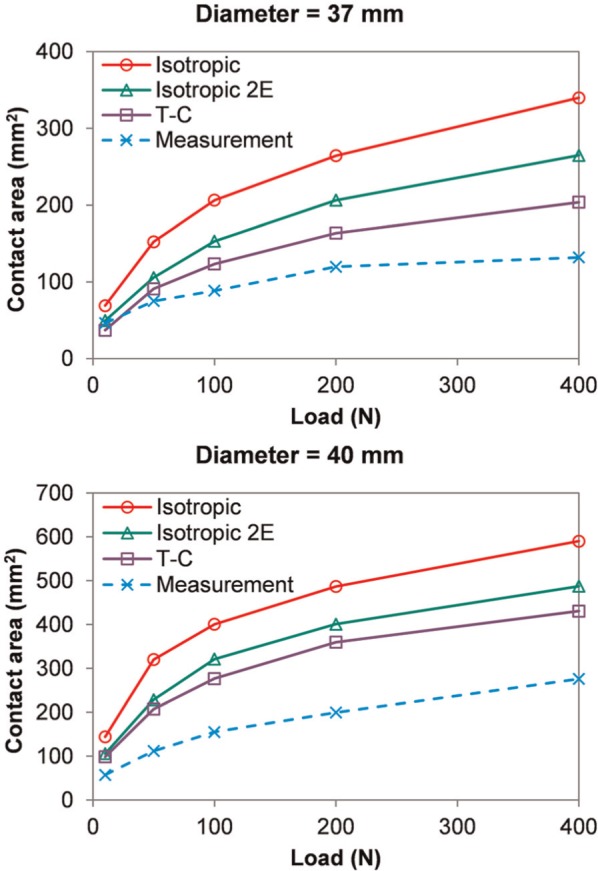
Contact area versus load of the FE models with different solid phase properties in comparison with the experimental measurements. T-C: tension–compression.

The magnitudes of contact area for the different head sizes are shown in Figure 6. Across the different loads investigated, the contact area of the T-C model was around 30%−50% lower than that of the isotropic model. The FE models with different solid phase properties generally predicted contact areas more than 30% higher than the values measured experimentally, but similar trends were seen across the loads investigated. The T-C model predicted areas closer to the experimental data than the isotropic model. However, when the aggregate modulus of the isotropic model was doubled, the contact area versus load behaviour was more comparable (<30% difference) to the T-C model as well as to the experimental measurement. It should be noted that in the stress contours of the FE models in Figures 4 and 5, the region with contact stress less than 10% of the peak value is represented by one colour, and thus, the area displayed by the other colours (>10% of the peak value) is smaller than the area of non-zero contact stress represented in Figure 6 in each case.

## Discussion

The aim of this study was to compare the FE hip model of a hemiarthroplasty, which incorporated the new biphasic modelling methodology with experimental measurements, and to assess the effect of different material models for the cartilage. As yet, there seem to have been only two studies that have reported direct validation of FE models of the hip contact conditions against experiments.^10,23^ Anderson et al.^23^ used an elastic model, while Pawaskar et al.^10^ used an ABAQUS biphasic model, which was unable to deliver solutions for the natural hip under physiological loading due to difficulties in obtaining convergence. In both these studies, the contact pressure and contact area were measured using pressure-sensitive films. However, such measurement techniques are open to question because the thickness (0.1–0.2 mm) and stiffness of such films may markedly alter the contact behaviour of the hip joint due to its highly congruent surfaces. To alleviate such potential artefacts, in this study, a staining fluid polymer was used to determine the contact area. However, other parameters such as contact pressure and fluid pressure cannot be predicted experimentally using this method, and this is a limitation.

The hemiarthroplasty model was chosen to validate the computational model so that different head sizes and radial clearances could be introduced, using two types of head with diameters of 37 and 40 mm. Loads of different magnitudes were applied. In addition, different properties for the solid phase of the cartilage were considered to evaluate the importance of the T-C constitutive relationship within the model.

Generally, the contact area predicted by both the isotropic and T-C FE models corresponded reasonably well to the experimental measurement. Both the shape and the location of the contact region predicted by the FE models were closely comparable to the experiment. The distinctive shape of the contact reflected the variation in the radius of curvature over the porcine acetabulum and indicates the importance of specimen-specific geometry, which corresponds to the conclusions made from previous studies of elastic models.^23,24^ The magnitudes of the contact areas in the FE models were higher than the experiments. This is likely to be because the fluid polymer may not make an imprint in regions of low contact stress, while the contact area in the FE models was calculated based on the region with non-zero contact stress. This would follow from the total joint replacement tests where the method underestimated the contact area compared to analytical calculations. It might also be because that the material property adopted from the literature is not subject-specific, or that the constitutive relationship considered in this study (i.e. isotropic or T-C) is not able to completely capture the tissue response. So far, however, it seems that no other measurement techniques allow more accurate prediction of the contact pattern within the very conforming hip joint. For both the isotropic and T-C models, similar trends in the magnitude of contact area to the experiment were observed over the loads investigated. Parameters other than the contact area were not mentioned in detail because they are not within the scope of this study.

In comparison to the experimentally measured area, a greater similarity in the shape and area of the contact was detected for the model with the T-C solid phase than the isotropic model. However, better agreement was also found between the T-C model and the isotropic model with doubled aggregate stiffness. This is because the expansion of the joint cartilage caused under compression was resisted by the higher tension stiffness in the T-C model, making the tissue stiffer than that in the standard isotropic model. These results suggest that during the early loading period within the joint, the stiffness of the cartilage is potentially underestimated by the isotropic constitutive relationship and can be represented more realistically by the T-C solid phase, which corresponds to the conclusions of previous studies using cylindrical models.^17,19^ However, the T-C model is still not able to account for the more complex behaviour of the tissue such as viscoelasticity of the solid matrix. The effects of further enhancement of the material model could be evaluated in future studies.

Although good agreement between the FE predictions and the experimental data was achieved, there are some limitations that should be mentioned. Apart from the potential measurement errors, only the instantaneous contact areas were evaluated without accounting for the time-dependent behaviour of the cartilage, because the variation in the contact area during the cartilage consolidation process within the hip is difficult to be accurately measured using the staining fluid polymer. However, the high similarity in the instantaneous results for a range of different input conditions provides some confidence in the model predictions.

For the experiment and the modelling, ideally identical boundary conditions would have been applied, but this was difficult to achieve in reality. In this study, the acetabular component in the FE model was oriented manually based on the bony landmarks. The similarity in the location of contact between the FE models and the experimental measurement that was obtained using this technique suggests it was adequate to locate the component. In addition, in the experiment, the cartilage was attached to the underlying bone which was supported by the cement, while in the FE model, the bone was assumed to be rigid. However, as found in a previous study,^14^ such an assumption is likely to have little influence on the model predictions.

In this study, the material properties of the cartilage were based on a previous curve-fitted test for cartilage from another porcine hip,^10^ and the tensile modulus in the T-C model was assumed to be 10 times higher than the aggregate modulus. Due to the potential variations in cartilage properties between different subjects, this simplification may potentially decrease the accuracy in the model predictions.

Owing to the high resolution of the scanned images (Figure 2), the geometric representation of the tissue can be obtained to a good level of accuracy. However, minimal errors in the model geometry may still exist due to the semi-automatic segmentation and smoothing techniques. According to the findings in a previous study,^14^ such variations in geometry are unlikely to greatly affect the accuracy of the models, yet the potential error due to this process should be evaluated more systematically.

In conclusion, in this first comparison of hip joint contact area between FE predictions made using a biphasic model in FEBio and experimental measurement, good agreement in the location and shape of contact was achieved, and a similar trend in the relationship between contact area and load was observed. A greater similarity in the results was obtained with the T-C solid phase, which, in terms of calculating the contact area, had similar effect to a stiffer isotropic model. The findings provide some confidence that the new biphasic methodology for modelling the cartilage is able to predict the contact mechanics of the hip joint. Future studies will seek validation for more parameters, investigate further the use of the T-C model, more effectively determine specimen-specific material properties and extend the studies to the human hip.
